# ASO Author Reflections: The Economic Promise of “Watch and Wait” in Rectal Cancer: Insights from a Systematic Review

**DOI:** 10.1245/s10434-024-16154-3

**Published:** 2024-09-03

**Authors:** Ishraq Murshed, Tarik Sammour

**Affiliations:** 1 Discipline of Surgery, Faculty of Health and Medical Sciences, Adelaide Medical School, University of Adelaide, Adelaide, SA Australia; 2 Colorectal Unit, Department of Surgery, Royal Adelaide Hospital, Adelaide, SA Australia

## Past

Rectal cancer is among the most prevalent cancers globally, imposing a substantial health care and economic burden. During recent decades, 5-year survival rates have improved due to advancements in early detection, accuracy of staging, refined surgical techniques, and progress in adjunct cancer therapies. However, global treatment costs for colorectal cancer are projected to escalate dramatically.^1^ Managing locally advanced rectal cancer (LARC) is particularly expensive given the frequent use of combination therapies and complex surgery.^2^ Non-operative management, or “watch and wait” (W&W), after a complete clinical response post-neoadjuvant therapy has been shown to offer disease-free survival comparable with that for surgery^3^ while improving patient quality of life (QoL).^4^ By avoiding surgery and inpatient hospitalization, W&W also presents the potential to be a more cost-effective approach.

## Present

This systematic review is the first to focus on the economic evaluations of W&W in LARC. The review, examining 12 studies from eight countries during an 8-year period (2016–2024),^5^ included seven cost-effectiveness analyses and five cost analyses, using both model- and trial-based methods. Despite notable variations in methodologic rigor and reporting quality, W&W consistently demonstrated cost-effectiveness and cost savings compared with surgery, from both health-system and patient perspectives, across diverse international health care settings.^5^

## Future

In an era of rising health care costs, W&W presents a unique opportunity to reduce expenditures while enhancing patient QoL. Although early evidence is promising, further research is essential to validate the findings and support the delivery of high-value, equitable health care. Although this systematic review suggests a favorable trend for W&W, definitive conclusions must be drawn cautiously due to the clinical heterogeneity and variable methodologic quality of the included studies. Future economic evaluations should adhere to best practice and reporting guidelines. Additionally, high-quality contemporaneous data on QoL for W&W patients would enable more precise health state utility assessments. The adoption of standardized comparators would further mitigate bias, and the inclusion of data from patients undergoing total neoadjuvant therapy is necessary to enhance the applicability of findings to current clinical practice.

## Data Availability

Not applicable.
